# Gene Clusters, Molecular Evolution and Disease: A Speculation

**DOI:** 10.2174/138920209787581271

**Published:** 2009-03

**Authors:** Leah I Elizondo, Paymaan Jafar-Nejad, J. Marietta Clewing, Cornelius F Boerkoel

**Affiliations:** 1 Interdepartmental Program in Cellular and Molecular Biology; 2 Department of Molecular and Human Genetics, Baylor College of Medicine, Houston, Texas; 3 Centre for Molecular Medicine and Therapeutics, Child and Family Research Institute, Department of Medical Genetics, University of British Columbia, Vancouver, Canada

## Abstract

Traditionally eukaryotic genes are considered independently expressed under the control of their promoters and cis-regulatory domains. However, recent studies in worms, flies, mice and humans have shown that genes co-habiting a chromatin domain or “genomic neighborhood” are frequently co-expressed. Often these co-expressed genes neither constitute part of an operon nor function within the same biological pathway. The mechanisms underlying the partitioning of the genome into transcriptional genomic neighborhoods are poorly defined. However, cross-species analyses find that the linkage among the co-expressed genes of these clusters is significantly conserved and that the expression patterns of genes within clusters have coevolved with the clusters. Such selection could be mediated by chromatin interactions with the nuclear matrix and long-range remodeling of chromatin structure. In the context of human disease, we propose that dysregulation of gene expression across genomic neighborhoods will cause highly pleiotropic diseases. Candidate genomic neighborhood diseases include the nuclear laminopathies, chromosomal translocations and genomic instability disorders, imprinting disorders of errant insulator function, syndromes from impaired cohesin complex assembly, as well as diseases of global covalent histone modifications and DNA methylation. The alteration of transcriptional genomic neighborhoods provides an exciting and novel model for studying epigenetic alterations as quantitative traits in complex common human diseases.

## INTRODUCTION

Gene expression is epigenetically regulated by DNA methylation, histone modifications, and chromatin remodeling [1]. The covalent modification of DNA and histones alters chromatin structure changing promoter accessibility to transcription factors. These DNA and histone modifications impose a level of transcriptional regulation in addition to the nuclear concentration and covalent modification of transcription factors. Therefore, in the context of a gene promoter, these DNA and histone modifications influence the binding of transcription factors and the regulation of gene expression [1].

Except for a few clusters of functionally related genes such as the rRNA, histone, Hox, and globin genes that are frequently co-regulated, the general perception has been that functionally unrelated genes are not co-regulated but are independently expressed under the control of their promoters and associated regulatory domains. However, in contrast to this perception, several recent studies in yeast, worms, flies, mice and humans have shown that genes co-habiting a chromatin domain or “genomic neighborhood” are frequently co-expressed [2-10]. Often these co-expressed genes do not function within the same biological pathway [10].

The mechanisms for partitioning the genome into clusters of co-expressed genes are poorly understood. However, the conservation of clustering across species suggests that this organization did not arise randomly but by selection and therefore perturbation of this organization could be deleterious. We review the evidence for co-expressed gene clusters, or genomic neighborhoods, discuss possible regulators of these clusters, and review a group of human disorders potentially disrupting genomic neighborhoods.

## GENOMIC NEIGHBORHOODS

Microarray, Serial Analysis of Gene Expression (SAGE) and Expressed-Sequence Tag (EST) analyses of gene expression have shown that many genes are organized into co-expressed clusters. Simplistically, these co-expressed gene clusters can be organized into three groups: 1) paralogous genes arising from gene duplications, 2) functionally related, non-duplicated genes, and 3) unrelated, non-duplicated genes that are co-expressed. Paralogous gene clusters contain genes that arose through tandem duplications resulting in genes that are often co-expressed as observed for duplicate gene clusters in *C. elegans* [5]. However, as illustrated by the clustered Hox genes, which are temporospatially co-regulated, not all clustered paralogous genes are necessarily co-expressed [11]. Clusters of functionally related genes include genes within the same pathway, genes encoding interacting proteins, or genes that affect the same trait. Not all functionally related genes are clustered within or among species. For example, genes within the same metabolic pathway, as defined by the Kyoto Encyclopedia of Genes and Genomes (KEGG) cluster in humans, whereas genes within the same Gene Ontology do not cluster in humans [12,  13]. Clusters of co-expressed unrelated, non-duplicated genes can be subdivided into those that are co-expressed at a fixed developmental time and/or location or into those that are co-expressed at all times and in all locations. For example, within the human genome, co-expression of housekeeping gene clusters has been found in addition to tissue-specific gene clustering of cartilage-expressed transcripts [14,  15].

### S. cerevisiae

Genomic clustering of co-expressed genes was first identified in studies of S. cerevisiae genes with cell-cycle dependent expression patterns [4]. About 25% of the genes expressed during the same phase of the cell cycle are arranged as pairs [16].

### C. elegans

Unlike most other eukaryotes, *C. elegans* has approximately 15 percent of its genes organized into operons, which are transcribed as polycistronic messages. These messages are subsequently processed into monocistronic mRNAs by trans-splicing [17]. Thus the genes within these operons are transcriptionally co-regulated. Additionally, tandem duplication of genes results in co-expression of many paralogues. Interestingly however, transcriptional co-expression is not limited to operons and tandem duplications because when the transcriptional data from such genes are excluded, the remaining genes are still transcriptionally clustered [5,  7,  9]. Also, some of this clustering reflects co-regulation of tissue-specific genes such as those expressed during spermatogenesis and in larval muscle [7,  9]. These observations support the idea that genomic neighborhoods are structural features that are, at least in part, independent of its occupants.

### Drosophila

In *Drosophila melanogaster*, about 20% of genes are arranged into clusters spanning 20 to 200 kb and containing 10 to 30 genes each [10]. Spellman and Rubin found that although paralogues and genes encoding functionally related products cluster, the gene composition of many other clusters is not defined by these identifiers [10].	In the transcriptional co-expression of adjacent genes observed by Boutanaev *et al.*, a third of testes-specific genes were expressed in clusters of two to six genes yet only about 16% of these clusters contained two or more paralogues [2]. Therefore, gene duplications account for many two-gene clusters but are not characteristic of clusters containing three or more co-expressed genes [2].

### Mouse

Profiling of murine testes-specific ESTs showed that the mouse genome contains clusters of genes that are specifically expressed in the testis [6]. As observed in *C.* *elegans* and *Drosophila*, nonparalogous genes co-expressed in the testes were organized into large clusters on all mouse chromosomes [6]. Similarly, the profiling of murine oocyte-specific ESTs identified oocyte-specific clusters of co-expressed genes adjacent to the telomeres of chromosomes 9, 12, 14 and 19 [8]. Because oocyte-specific genes outside of these clusters were not biased toward a telomeric location, the authors suggested that the oocyte-specific telomeric clusters were nonrandom [8]. Additionally they suggested that this organization facilitates silencing of oocyte-specific genes in non-ovarian tissues because murine telomeres were highly heterochromatic [8]. Therefore these oocyte-specific clusters may constitute genomic neighborhoods that are regulated and controlled in a tissue-specific manner by higher order genomic organization.

### Human

In humans, highly expressed genes cluster in large domains called RIDGEs (Regions of IncreaseD Gene Expression) and, interestingly, 40-50% of these RIDGEs map to telomeres [3]. Caron *et al.* observed that chromosomes with high gene density contain an increased number of RIDGEs when compared to chromosomes with low gene density [3]. More specifically, RIDGEs themselves have high gene and short interspersed nuclear repetitive DNA element (SINE) density and high GC content [3,  18].

Examining human Serial Analysis of Gene Expression (SAGE) data from 14 different types of tissues, Lercher *et al.* identified co-expression clusters of highly expressed housekeeping genes but not of tissue-specific genes [14]. Similar to RIDGEs, these clusters were highly correlated with regions of high GC content [18,  19]. Based on these observations, they hypothesized that housekeeping genes would be clustered in open chromatin to facilitate transcription. Consistent with this, they found that highly expressed gene clusters associated cytogenetically with the lightest staining Giemsa bands, i.e., relaxed chromatin [19].

While mapping cartilage-expressed transcripts (CETs) in the human genome, Yager *et al.* was able to identify clustering of co-expressed non-housekeeping genes Moreover, it has recently been shown that human embryonic stem cells contain co-expression domains that are distinguishable from co-expression domains identified in the embryoid body [20]. These data suggest that not all clusters of co-expressed genes consist of housekeeping genes, but rather some clusters also constitute tissue-specific genes, or even differentiation-specific genes.

Further refining the co-expression clusters within the human genome, Sémon and Duret found that ~65% of the human genes belong to co-expressed gene clusters and that the distribution of cluster size, as judged by the number of genes, is biased toward small clusters [21]. The clusters that they defined also were not limited to functionally related or housekeeping genes.

In summary, clustering of co-expressed genes occurs with each species and across species [12,  21-23]. Interestingly, this is not only applicable to paralogues, functionally related genes, tissue-specific genes, or housekeeping genes but also apparently to unrelated genes. Studies in several species show that clusters of co-expressed genes fall within genomic regions containing low recombination rates, demonstrate coevolution of gene expression with gene clustering, and conservation of clusters between species. These observations suggest that these co-expressed clusters are maintained by natural selection [24-26]. Therefore, if the assembly of co-expressed genes into clusters is nonrandom, then this organization must be highly regulated within the three-dimensional structure of the nucleus, and one could hypothesize that the loss of this organization would be deleterious to the organism.

## THREE-DIMENSIONAL NUCLEAR ORGANIZATION AND GENE REGULATION

Although the mechanisms responsible for partitioning the genome into regions of co-expressed clusters are incompletely defined, our current understanding of the three-dimensional organization of the nucleus suggests possible candidate mechanisms. Factors contributing to the formation of genomic neighborhoods likely include chromosomal spatial orientation, chromatin interaction with the nuclear lamina, and the association of chromatin with subnuclear structures.

Oliver *et al.* proposed three models for co-expression of non-paralogous genes: 1) the incidental expression model, 2) the chromatin domain model and 3) the three-dimensional space model [27]. The incidental expression model predicts that when a transcription factor binds a target gene, it also incidentally activates neighboring genes and that this incidental co-expression of neighboring genes may have little biological relevance. The structural domain model proposes that the opening of an entire genomic neighborhood facilitates the binding of the transcriptional machinery to any gene within the neighborhood and that these neighborhoods would be demarcated and regulated by boundaries such as insulator complexes. Finally, according to the three-dimensional space model, a target gene is recruited to a subnuclear location such as a transcription factory and adjacent genes would also be exposed to the transcription machinery and therefore also transcribed [27]. Each of these non-exclusive models emphasizes a different aspect of nuclear architecture and thus provides a slightly different mechanistic view of the role for nuclear architecture in regulation of genomic neighborhoods.

### Nuclear Envelope

The lamins and associated proteins are the major components of the nuclear lamina. These proteins maintain the integrity of the nuclear envelope [28], provide a structural attachment point for chromatin [29], help define DNA replication sites [28,  30-32], localize nuclear bodies [33], and facilitate transcription [34-37]. Spann *et al.* showed that interfering with lamin organization inhibited RNA polymerase II activity and therefore suggested that the nuclear lamina is required for the assembly of basal transcription factors and RNA polymerase II [36]. A role for the nuclear lamina in gene expression is further substantiated by interaction of lamin-associated proteins with the transcriptional apparatus. For example, emerin, an inner nuclear envelope protein, not only binds lamins A and B but also interacts with the transcription factors GCL, Btf, Lmo7, BAF, and YT521-B [34,  37-41]. The roles of the nuclear lamina in defining regions of transcriptional co-expression have not been reported. However, as described below, the pleiotropic disorders associated with mutations of the nuclear lamina suggest that it has both global and tissue-specific effects on gene expression.

### Nuclear Genomic Organization

Rabl first proposed a territorial organization of interphase chromosomes in 1885 [42]. Interphase nuclei have regions of high density (heterochromatin), regions of lower density (euchromatin), and regions of lowest density (nucleoli) [43]. However, the DNA from the various chromosomes is not randomly intertwined, rather chromosomes occupy non-overlapping territories of irregular shape [44-48]. There are two existing models for the distribution of chromosome territories [49]. One model suggests that gene-dense chromosomes are located more internally than gene-poor ones. Although not consistently observed [50], this arrangement has been frequently reported for mammalian and chicken cells [46,  51,  52] as well as for homologous chromosomes in higher primates [53] and syntenic chromosomes in humans and mice [54,  55]. The other model proposes that chromosomal territories are organized by gene function. For example, chromosomal organization arises from interactions between chromosome territories or domains required for DNA repair and homologous recombination [49,  56].

Development-specific and cell cycle-specific spatial alignment of homologous chromosome segments occurs in *Drosophila* [57-59] and for mammalian chromosomes in a variety of cells [60,  61]. Interestingly, the positioning of interphase chromosomes is largely inherited from mother to daughter nuclei in mammals suggesting that this may be a high order epigenetic regulatory mechanism [62-66].

### Subnuclear Structures and the Interchromosomal Domains

Chromatin from separate territories minimally intermingles [46,  67]. Electron microscopy and polymerization of probes such as vimentin clearly define an interchromosomal domain or space between chromosomal territiories [68-72]. Nuclear bodies, such as Cajal and PML bodies, speckles, and specific nascent RNA accumulations lie in the interchromosomal domains along the surface of chromosome territories and are excluded from the chromosome territories [69,  72-75]. Genes are generally distributed along the periphery of chromosome territories and invaginating channels where they can loop out into interchromosomal domains upon the induction of expression [54,  55,  76-80]. Thus, transcription may predominate along the periphery of a chromosome territory since this would allow access for transcription factors and facilitate processing and transport of mRNA [46,  71,  72,  77]. Moreover, these findings suggest that the topology of genes relative to the interchromosomal domain compartment might provide a framework for the clustering of co-expressed genes and contribute to the overall regulation of gene expression [46,  67,  71,  72,  77,  81,  82].

### Boundary Elements

Observations from yeast, *Drosophila*, chicken, mouse and human studies show that insulators compartmentalize the genome into separate regions of gene expression through interactions with DNA, the nuclear matrix, and other protein components [83-85]. Through these interactions, insulator complexes are proposed to facilitate the formation of higher order genomic structures that can be independently regulated and are defined by two activities: 1) they can inhibit the spread of heterochromatin, and/or 2) they can block a transcriptional enhancer from activating a promoter when located between the two [85].

One of the best-studied insulators is the *gypsy* element found in the *Drosophila gypsy* retrotransposon. Proteins that directly or indirectly bind the *gypsy* insulator include suppressor of hairy wing, modifier of mdg4, Centrosomal protein 190 kD, Trithorax-like, and components of the nuclear matrix [86- 89]. Modulation of the interaction between these proteins and the gypsy insulator sequences defines higher order chromatin structures, such as chromatin loops, and the functionality of each gypsy sequence [86,  87,  89].

In vertebrates, our understanding of insulator function primarily comes from studies of imprinted genomic domains, such as the Beckwith Weideman region, and the beta-globin gene cluster [90,  91]. CCCTC-binding factor (CTCF), which binds the insulator sequences in these regions as well as many other sites, is the best-characterized mammalian insulator-binding protein. The 5’ CHS4 insulator sequence, which is located upstream of the beta-globin cluster, has two separable functions: 1) it inhibits the adjacent heterochromatin from spreading into the beta-globin locus, and 2) it blocks enhancer-promoter interactions [92]. In addition to binding sequences such as CHS4, CTCF binds nucleophosmin, a nucleolar protein, and components of the nuclear matrix [93-95]. Binding of CTCF to the CHS4 and other insulators is proposed to facilitate the formation of chromatin loops and tether these loops to subnuclear structures, such as the nucleolus and the nuclear matrix [95].

Another chromatin structural motif that is potentially involved in the establishment of genomic neighborhoods is the cohesin complex. Cohesin complexes were originally identified for their role in maintaining sister chromatid cohesion prior to their separation during anaphase [96]. The *Drosophila* protein Nipped-B loads the cohesin complex onto the chromosomes and is also required for facilitating enhancer-promoter communication [97-99] and regulating gene expression [97]. This suggests that cohesin complexes may also be structural elements defining and regulating gene co-expression domains and that impaired or errant localization of cohesin complexes could affect RNA transcription across large genomic regions.

In summary, the three-dimensional structure of the nucleus provides a context for understanding and elucidating transcriptional genomic neighborhoods and an experimental basis for understanding the incidental expression, chromatin domain and three-dimensional space models for clustering of co-expressed genes. The intricate architecture of the nucleus and the nonrandomness of genomic neighborhoods suggest that perturbations at many different levels could cause dysregulation of genomic neighborhoods. This would result in altered spatial localization of chromatin within the nucleus and ultimately predispose to diseases that could affect multiple organ systems.

## DEREGULATION OF GENOMIC ORGANIZATION AND DISEASE

To date, most epigenetic research in human disease has focused on the histone and DNA modifications occurring at discrete gene loci. Increasingly, however, genome-wide alterations are being identified in human cancers and in rare Mendelian disorders. These disorders affect gene expression through disruption of higher order genomic organization, and we review a set of diseases illustrating this. We have grouped the disorders in a hierarchy extending from three-dimensional intramolecular positioning of chromosomes to those resulting in molecular alterations of DNA across genomic neighborhoods.

### Chromosomal Rearrangements: Disorders of Chromatin Localization?

As reviewed above, several studies have suggested that there are nuclear territories specific for each chromosome within a given cell type [49,  100-104]. Additionally, studies have shown that there are specific transcriptional regions or neighborhoods within the nucleus [46,  105]. These two observations suggest that reciprocal translocation of chromosomal segments would alter gene expression by placing the gene in a different transcriptional environment within the nucleus. Thus, the pathology arising from the translocations is not limited to disrupting genes, separating genes from *cis* regulatory elements, or placing genes within a new *cis* chromatin environment [106-109] but could also arise by changing the position of a gene or group of genes relative to the interchromosomal domain compartment and associated transcription factors. To date, this model has not been extensively investigated in somatic or germline human diseases associated with chromosomal rearrangements and genome instability.

### Nuclear Envelopathies: Disorders of Nuclear Envelop Integrity and Maintenance of Chromosomal and Interchromosomal Domains?

The lamins and associated proteins constitute major components of the nuclear lamina and are involved in maintaining the integrity of the nuclear envelope [28], providing a structural attachment point for chromatin [29], defining DNA replication sites [28,  30-32], localizing nuclear bodies [33], and mediating transcription [34-37]. Based on these identified functions, disruption of the nuclear matrix would impair the ability of chromatin to attach to the nuclear lamina and thereby hinder the ability of a cell to define and to maintain the domains necessary for appropriate gene expression and genomic stability. Mutations of nuclear lamina proteins have been identified in many different diseases including Emery-Dreifuss muscular dystrophy (OMIM #310300, #181350, #604929), dilated cardiomyopathy (OMIM #115200), Charcot-Marie-Tooth disease (OMIM #605588), mandibuloacral dysplasia (OMIM #248370, #608612), Dunnigan-type familial partial lipodystrophy (OMIM #151660), Hutchinson-Gilford progeria (OMIM #176670), atypical Werner syndrome (OMIM #277700), Pelger-Huët anomaly (OMIM #169400), Greenberg skeletal dysplasia (OMIM #215140), restrictive dermopathy (OMIM #275210), melorheostosis (OMIM #155950), Buschke-Ollendorff syndrome (OMIM #166700), and lipoatrophy with diabetes, hepatic steatosis, hypertrophic cardiomyopathy, and leukomelanodermic papules (OMIM #608056) [110].

Given the role the nuclear lamina plays in regulating transcription [34-37], we propose that disruption of the nuclear lamina might affect transcription by altering the nuclear organization defining genomic neighborhoods. Consistent with this hypothesis, studies of gene expression in mandibuloacral dysplasia and Emery-Dreifuss muscular dystrophy were consistent with a deregulation of gene expression at multiple loci [111-113]. Therefore, superimposed on the impaired DNA repair and nuclear envelope stability, alterations in transcription help explain the pleiotropy, variable penetrance, and variable expressivity of diseases associated with mutant lamina proteins.

### Schimke Immuno-Osseous Dysplasia: a Disorder of Genomic Neighborhood Establishment and Maintenance?

Mutations in *SMARCAL1* (SWI/SNF-related, matrix-associated, actin-dependent regulator of chromatin, subfamily A-like 1) cause Schimke immuno-osseous dysplasia (SIOD; OMIM 242900). SIOD is an autosomal recessive disorder of T-cell immunodeficiency, spondyloepiphyseal dysplasia, renal failure, hypothyroidism, episodic cerebral ischemia, and bone marrow failure. Both clinical and cell culture studies suggest that functional SMARCAL1 protein is required for the proliferation of many of the affected tissues [114-116]. Consistent with its role as an annealing DNA helicase [117], the SMARCAL1 protein binds nucleosomes and has DNA-dependent, RNA-independent, ATPase activity [118,  119]. The SMARCAL1 protein is predominantly localized to the open chromatin and loss of functional SMARCAL1 apparently causes cell autonomous disease by altering chromatin helical torsion [117,  120]. In SMARCAL1 mutant tissues, transcription is altered across entire genomic neighborhoods suggesting that SMARCAL1 is necessary either for the establishment or maintenance of transcriptional genomic neighborhoods [Boerkoel, CF, and co-workers, unpublished results].

### Cornelia de Lange Syndrome: A Disorder of Cohesin-Regulated Transcription?

Mutations in *NIPBL* (Nipped-B-Like) results in Cornelia de Lange syndrome (CDLS: OMIM 122470). CDLS is an autosomal dominant multisystem disease characterized by distinctive facial features, growth retardation, hirsutism, upper limb reduction defects, cognitive impairment, and behavioral abnormalities as well as other congenital malformations. In *Saccharomyces cerevisiae, S. pombe* and *Xenopus*, the *NIPBL* homologues (*Scc2*, *Mis4* and *Xscc2*), known collectively as adherins, load the cohesin protein complex onto chromosomes [121-125] and are required for sister chromatid cohesion. As described above, the *Drosophila* *NIPBL* homologue, *Nipped-B*, is not only required for sister chromatid cohesion but also for facilitating enhancer-promoter communication [97-99]. This latter observation suggests that the pathology of CDLS is not only due to precocious sister chromatid separation [126] but also due to aberrant transcription. Since the cohesin complex binds approximately every 10kb along the *S. cerevisiae and S. pombe *interphase chromosome [127, 128], impaired or errant localization of cohesin complexes have the potential to profoundly affect RNA transcription across large genomic regions.

### Roberts Syndrome: A Disorder of Cohesin-Regulated Transcription?

Mutations in *ESCO2* (Establishment of Cohesion 2), which encodes an acetyltransferase, has been shown to cause Roberts syndrome (RBS: OMIM 268300) and SC phocomelia (OMIM 269000). RBS is an autosomal recessive disease characterized by hypomelia, growth deficiency, craniofacial anomalies, microcephaly, and mental deficiency. Severely affected infants may be stillborn or die shortly after birth. SC phocomelia, a milder disorder, has less limb reduction and includes flexion contractures, midfacial hemangioma, hypoplastic cartilage of ears and nose, scant silvery-blond hair, and cloudy corneae. In *S. cerevisiae, S. pombe, Drosophila* and *H. sapiens*, the *ESCO2* homologues are required for sister chromatid cohesion [129-133] and are bound to chromosomes throughout the cell cycle [129, 130]. In addition to affecting mitotic progression and ploidy [134], we hypothesize that, like Cornelia de Lange syndrome, mutations of ESCO2 could give rise to aberrant transcription across large genomic regions and that this partially accounts for the pathology of RBS and SC phocomelia.

### Rubenstein Taybi syndrome: A Local or Global Disorder of Histone Acetylation?

Mutations in *CREBBP* (cAMP-responsive element binding protein (CREB) binding protein) and in *EP300* (e1a-binding protein p300) cause Rubinstein Taybi syndrome (RTS: OMIM 180849). RTS is an autosomal dominant disorder of growth retardation, facial abnormalities, broad thumbs and toes, and mental retardation. As histone acetylases, CREBBP and EP300 promote the decondensation of chromatin and interact with many transcription factors to facilitate RNA transcription [135-136]. More specifically, CREBBP-mediated histone acetylation plays a role in synaptic plasticity, long term memory and in ameliorating neuronal degeneration [137-140]. This can partially explain the mental retardation seen in RTS, but the mechanisms by which decreased CREBBP dosage causes malformations remain obscure. If CREBBP and EP300 are generally involved in facilitating the transcription of many or most genes, then decreased dosage of CREBBP and EP300 could globally impair the RNA transcription including that of genomic neighborhoods.

### ICF Syndrome: A Global Disorder of DNA Methylation?

Mutations in *DNMT3B* (DNA methyltransferase 3B) cause immunodeficiency-centromeric instability-facial anomalies syndrome (ICF: OMIM 242860). ICF syndrome is an autosomal recessive disorder of genomic methylation that mainly affects satellites 2 and 3 of constitutive heterochromatin, although centromeric alpha satellites [141], Alu sequences [142, 143], D4Z4 and NBL2 repeats [144], and certain imprinted genes [145, 146] are also involved. In addition, the inactive X chromosome (Xi), which consists of facultative heterochromatin, is globally undermethylated although all sequences are not equally affected in all patients [143-147]. Interestingly, Xi CpG island demethylation is not accompanied by significant biallelic transcriptional reactivation [142, 148], and consistent with this observation, histone modifications along the Xi are normal [149].

Although there has not been an extensive analysis of the transcriptome of ICF patients, several considerations suggest that the pathology of ICF may be partially caused by large-scale transcriptional alterations: 1) genomic instability translocates chromosomal fragments to inappropriate chromosome territories; 2) loss of repetitive DNA methylation impairs heterochromatin formation which is a key long-range transcriptional regulator; and 3) methylation of repetitive elements plays a key role in the regulation of chromatin boundary elements that modulate transcription.

### Rett Syndrome: A Disorder of Methylated DNA Recognition or of RNA Processing?

Mutations in *MECP2* cause a spectrum of disorders including Rett syndrome (OMIM 312750), neonatal-onset encephalopathy, mental retardation, autism, and an Angelman-like syndrome [150]. MECP2 binds methylated promoter sites and recruits the mSinA/Histone Deacetylase (HDAC) 1, 2 corepressor complex [151]; this process mediates dynamic repression of gene expression [152, 153]. These data and transcriptome analyses suggest that the pathology associated with MECP2 mutations results from inappropriate gene expression [154, 155]. Consistent with this hypothesis, some patients with mutations in *MECP2* exhibit histone H4 hyperacetylation [156] and dysregulation of alternative splicing of some genes [157]. Thus loss of functional MECP2 appears to mediate widespread alterations in gene expression at both the level of RNA transcription and mRNA processing. If MECP2 were necessary to facilitate the interaction of chromatin with the nuclear matrix, then such dysfunction could arise through altered proximity of genes to the interchromosomal domains as well as through defects in the ability to form heterochromatin.

### Cancer: A Panoply of Disorders Affecting All Levels of Genomic Organization?

Cancerous tissue and tumor-derived cell lines exhibit errors at nearly all levels of nuclear organization including chromosomal aberrations [158, 159], altered nucleosome spacing [109], covalent histone modifications [160], and DNA methylation [161]. Besides the established roles in loss of heterozygosity for tumor suppressor genes and oncogene activation, we propose that chromosomal rearrangements might also play a role in cancer by altering the expression of genes across large domains by relocating them to a different chromosomal territory.

The cancer-associated ATP-dependent chromatin remodeling enzymes *SMARCB1* (SWI/SNF-related, matrix-associated, actin-dependent regulator of chromatin, subfamily B, member 1) and *BRG1* (brahma-related gene 1), and *BRM* (brahma) regulate gene expression by altering nucleosome spacing [162-166]. Transcriptome analyses of tumors with dysfunction of *SMARCB1, BRG1, *and* BRM* will clarify whether transcriptional changes globally affect genomic neighborhoods or are targeted to specific genes.

Abnormalities of global and targeted histone acetylation occur in several human neoplasms. In acute promylelocytic leukemia, acute lymphocytic leukemia, and non-Hodgkins lymphoma, mutations of proteins that recruit HDACs to DNA errantly activate and target interacting HDACs [162]. In several human cancers, this results in a reduction of overall histone acetylation [167]. In addition, histone acetylases are deregulated in some human neoplasias; this dysregulation plays a crucial role in tumor development and progression [168-171]. Underscoring the role of histone acetylation in cancer, HDAC inhibitors slow tumor cell proliferation and induce differentiation. These changes are associated with global affects on transcription [172, 173] and suggest that the tumor phenotype results from the altered expression of many genes.

Human tumor DNA is globally hypomethylated [174, 175]. Malignant cells can have 20%–60% less genomic 5-methyl-cytosine than normal counterpart cells [175]. However, genomic hypomethylation does not associate with overexpression of oncogenes. Rather three mechanisms have been invoked for the contribution of global DNA hypomethylation to carcinogenesis: chromosomal instability, reactivation of transposable elements, and loss of imprinting [176-179]. Each of these processes is involved in regulation of chromatin structure and likely in defining transcriptional genomic neighborhoods. Additionally, in contrast to the global hypomethylation, CpG islands in the promoter regions of many tumor suppressor genes undergo hypermethylation in cancer cells leading to gene silencing [180, 181], and the number of hypermethylated tumor suppressor genes increases with the malignant potential [181-184]. Thus at the level of DNA methylation, the cancer phenotype appears to be a combination of global chromatin structural changes through overall DNA hypomethylation and of targeted gene inactivation through promoter hypermethylation.

## GENOMIC ORGANIZATION AND NATURAL SELECTION

The correlation of perturbed genomic neighborhoods with disease suggests that they are functionally significant and not purely coincidental. This hypothesis is also supported by several lines of evidence that these clusters arose and are preserved by natural selection. First, co-expression clusters contain fewer chromosomal breakpoints between human and mouse than expected by chance [26]. Second, highly co-expressed clusters of genes are phylogenetically conserved between human and mouse and consist mainly of nonparalogous genes [185, 186]. Third, comparison of the human and chicken genomes shows that there is more linkage retention among genes within clusters than outside of clusters [21]. Fourth, analysis of coexpression of neighboring genes within the mouse and human genomes suggests that there is coevolution of the pattern of expression in neighboring genes [21]. Fifth, genes within conserved regions of synteny between *Drosophila melanogaster *and *Drosophila pseudoobscura* have highly correlated expression patterns [187].

## CONCLUSIONS

Advances in our understanding of gene expression and distribution in the genome make the supposition of random gene order in eukaryotes increasingly untenable. Recent advances in nuclear biology have not only initiated novel views of the role of genomic organization in gene expression regulation but also new insights into the cause of disease.

The nonrandom organization of the genome into a series of chromosomal blocks that are transcriptionally regulated provides possible insight into some recent unexplained observations during development. For example, during differentiation of hematopoietic stem cells, particular chromosomal regions are inactivated [188], and during differentiation of non-neuronal tissues, specific chromosomal intervals are transcriptionally down-regulated [189].

According to this model of genomic organization, the pathophysiology underlying many diseases could arise as a result of alterations in genomic organization causing quantitative transcriptional disturbances. The cumulative effect of these transcriptional disturbances or the confluence of aberrant transcription of several genes would then sufficiently perturb homeostasis to predispose to or cause disease. Thus, for the rare Mendelian disorders considered above, the chromosomal aberration or mutant protein could effect the disease phenotype by causing quantitative changes in gene transcription through altering the nuclear localization or structure of large chromatin domains. The variable expressivity of many of these disorders reflects the quantitative differences in transcription, and the phenotypic features can be considered quantitative traits.

Considering the features of these rare Mendelian disorders as quantitative traits provides a model for the variable expressivity and pleiotropism; characteristics which cannot be explained simply by dysregulation of a single pathway or gene interaction network. It also explains why segregation of gene mutations for these diseases frequently predispose to rather than cause disease and why many features of these diseases have genocopies. Lastly, since genomic neighborhoods are likely regulated by both genetic and epigenetic mechanisms, this model integrates both in the causation of human diseases.

Viewing co-expression of clustered genes as a quantitative trait also provides an explanation of why genomic neighborhoods would be a substrate for and not only a product of natural selection. In contrast to strong single gene mutations that result in dramatic changes in fitness, alterations in expression of genes, and likely gene clusters, allow gradual and persistent selection of offspring that are more fit than their parents [190]. Moreover, since mRNA levels for many genes are heritable [191], gene expression and co-expressed gene clusters could be the subject of selection over many generations.

In this context, the features of these rare Mendelian disorders provide a novel method for modeling and studying quantitative traits and multifactorial disorders. Such disorders include mental retardation, diabetes, hypertension, atherosclerosis, vascular cognitive impairment, and Metabolic Syndrome (type 2 diabetes, cardiovascular disease, and hypertension). Schimke immuno-osseous dysplasia (SIOD) nicely illustrates this for atherosclerosis and vascular cognitive impairment. Approximately half of SIOD patients develop vascular cognitive impairment secondary to atherosclerosis. Since mutations of SMARCAL1 alter transcription across chromatin domains containing many genes with altered transcription in atherosclerotic plaques, we hypothesize that the confluence of these multiple transcriptional changes predisposes to atherosclerosis. However, the degree of predisposition is dependent on the level of transcriptional alteration, and the development of atherosclerosis results from the interaction of these transcriptional changes with environmental, genetic, and epigenetic factors. Therefore as a model for generic atherosclerosis, the regions of altered transcription in SIOD are quantitative trait loci for atherosclerosis. Intersection of these loci with genetically identified disease loci further delineate the genes and pathways contributing not only to rare Mendelian disorders but also gives insight into the pathomechanism of complex diseases.

## FUTURE DIRECTIONS

Defining better the selective pressure for and on genomic neighborhoods and determining whether transcriptional alterations of these are a common cause of complex disorders requires further testing. These future studies will provide an exciting opportunity to define the heritable epigenetic variation contributing to complex diseases and address many of the questions regarding the contribution of epigenetic variation to quantitative traits. Additionally, these studies will allow investigators to clarify whether the increasing incidences of diseases such as asthma, Metabolic Syndrome, and some neoplasias are due in part from increasing environmental influences on epigenetic traits. Answering these questions is essential for defining the contribution of genomic organization in human disease.
